# Losartan Ameliorates Coronary Neointimal Thickening in a Mouse Model of Kawasaki Disease: A Pilot Study

**DOI:** 10.3390/biomedicines14071646

**Published:** 2026-07-22

**Authors:** Eisuke Suganuma, Satoko Honda, Rina Umiguchi, Sayaka Ishikawa, Ayako Shimamura, Marina Tanaka, Masashi Kyushiki, Atsuko Nakazawa

**Affiliations:** 1 Division of Infectious Diseases and Immunology, Saitama Children’s Medical Center, 2-1 Shintoshin Chuou-ku, Saitama 330-8777, Japan; 2 Division of Clinical Research, Saitama Children’s Medical Center, Saitama 330-8777, Japan

**Keywords:** angiotensin receptor blocker, *Lactobacillus casei* cell wall extract, neointima, coronary stenosis, vascular smooth muscle cell, elastin degradation

## Abstract

**Background:** Patients with Kawasaki disease (KD) who develop coronary artery aneurysms (CAAs) are at increased risk of future fatal coronary events. Pharmacotherapeutic strategies to prevent coronary stenosis are still lacking. In this exploratory study, the therapeutic effect of the angiotensin receptor blocker (ARB) losartan on coronary artery (CA) stenosis was investigated in a murine model. **Methods:** Five-week-old male C57BL/6J mice were intraperitoneally injected with 1000 μg of *Lactobacillus casei* cell wall extract (LCWE) (*n* = 12) to induce CA stenosis. Two weeks later, the LCWE-injected mice (*n* = 12) were divided into two groups: six received drinking water containing losartan (100 mg/L) (LCWE+ARB), while six received normal drinking water (LCWE group). A control group (*n* = 5) received phosphate-buffered saline (PBS) instead of LCWE. Sixteen weeks after LCWE administration—corresponding to the peak of CA stenosis and 14 weeks after treatment initiation—the mice were euthanized for histological evaluation of the coronary arteries. **Results:** Losartan treatment significantly reduced the coronary arteritis score (median [IQR (interquartile range)]: 0 [0–9.5] vs. 21.5 [15–24.3], *p* = 0.003). LCWE-induced neointimal formation with vascular smooth muscle cell proliferation and subsequent CA stenosis were markedly attenuated in losartan-treated mice (25% vs. 100%, *p* < 0.001). Losartan-associated attenuation of CA stenosis was accompanied by the preservation of medial calponin expression, a reduction in the number of proliferating cell nuclear antigen (PCNA)-positive cells in the neointima, and a decrease in serum MMP-9 (matrix metalloproteinase-9) levels. **Conclusions:** These findings from a murine model of KD provide preliminary evidence that losartan may attenuate coronary artery remodeling. Further mechanistic studies are warranted to clarify its potential translational relevance.

## 1. Introduction

Kawasaki disease (KD) is an acute febrile illness of unknown etiology that primarily affects infants and young children; it was first described by Tomisaku Kawasaki in 1967 [1]. The most serious complication is coronary artery aneurysm (CAA). According to the latest nationwide epidemiological survey of KD in Japan, the incidence of CAA has decreased to approximately 2–3% with the availability of multiple therapeutic options, including intravenous immunoglobulin (IVIG) [2], corticosteroids such as prednisolone (PSL) [3], infliximab [4], cyclosporine A [5], and plasma exchange therapy [6]. However, children with CAAs are at increased risk for serious coronary events, particularly thrombosis, coronary stenosis, myocardial infarction, and even sudden death [7]. Autopsy studies have reported various vascular wall changes associated with CAA, including intimal thickening, medial destruction, and calcification [8]. Endothelial dysfunction is recognized as an important contributor to the development and progression of KD vasculopathy [9]. Persistent endothelial dysfunction promotes vascular inflammation, leukocyte recruitment, and vascular remodeling, ultimately contributing to CA abnormalities and stenotic lesions [10]. However, pharmacological strategies targeting chronic coronary vascular remodeling remain limited.

Angiotensin receptor blockers (ARBs) are widely used to prevent cardiovascular events such as myocardial infarction in patients with hypertension, heart failure, and atherosclerosis [11,12,13]. Basic research has demonstrated that ARBs have a wide range of beneficial effects beyond blood pressure reduction. These include the preservation of vascular endothelial function [14], anti-inflammatory [15] and antioxidant effects [16], and the inhibition of vascular smooth muscle cell (VSMC) proliferation [17]. CAAs associated with KD are also thought to share many similarities with cardiovascular diseases, including atherosclerosis and aneurysms, in that the structure of the vessel wall is destroyed by panvasculitis. In KD, CAAs are associated with an increased long-term risk of fatal or nonfatal coronary events [18]. Beyond aneurysm formation itself, progressive localized stenosis has been observed during follow-up, often at the inlet or outlet of aneurysmal segments [19,20]. These findings suggest that active vascular remodeling, rather than the aneurysm alone, underlies the development of coronary obstruction. These findings support further investigations into the mechanisms of neointimal formation and stenosis in KD-related coronary vasculopathy.

*Lactobacillus casei* cell wall extract (LCWE)-induced vasculitis, first described by Lehman et al. in 1985, is a KD-like murine vasculitis model in which a single intraperitoneal administration of LCWE induces severe vasculitis involving the aorta and the origins of the coronary arteries [21]. Our previous experimental studies on ARBs in LCWE-induced vasculitis focused primarily on their anti-inflammatory effects during the acute phase of coronary arteritis, including the suppression of coronary perivasculitis and myocarditis [15]. Furthermore, our clinical study suggested that combination therapy with ARBs or angiotensin-converting enzyme inhibitors (ACEis) may be associated with the regression of medium- and large-sized CAAs in patients with KD during long-term follow-up [22]. However, the mechanisms underlying these beneficial vascular effects remain incompletely understood.

One major obstacle in this field has been the lack of an appropriate animal model that reproduces chronic coronary vascular remodeling, particularly stenotic lesions following vasculitis. To address this issue, we previously established a novel murine model in which intraperitoneal injection of LCWE induces severe coronary arteritis followed by progressive CA stenosis secondary to neointimal formation [23]. On the basis of these findings, in the present study, the effects of ARB therapy on vasculitis-related CA stenosis were investigated using this novel murine model, with a particular focus on the characteristics of VSMC and neointimal remodeling. Unlike previous studies that focused primarily on acute inflammatory changes, the present study focused on the effects of ARB treatment on chronic CA remodeling and stenotic lesions following vasculitis.

## 2. Materials and Methods

### 2.1. Animals

Four-week-old male C57BL/6J mice were purchased from CLEA Japan (Tokyo, Japan) and were allowed to acclimate to the research environment for 1 week prior to experimentation. The mice were housed under specific pathogen-free conditions in groups of 3 per cage and maintained under a 12 h light/dark cycle at an ambient temperature of 23–25 °C and a relative humidity of 45–55%. The mice were fed a standard irradiated laboratory rodent diet (CE-2; CLEA Japan Inc., Tokyo, Japan), a breeding diet formulated for mice, rats, and hamsters and sterilized by gamma irradiation (30 kGy), with food and water provided ad libitum. According to the manufacturer, CE-2 is a GLP-compliant standard rodent diet consisting mainly of vegetable protein, with a balanced inclusion of animal protein. All the experimental procedures were conducted in accordance with the institutional guidelines and regulations of Saitama Children’s Medical Center, Japan. The study protocol was approved by the Animal Experimental Ethics Committee of Saitama Children’s Medical Center (approval numbers: 2020-003 and 2021-003). All experiments were performed in compliance with the ARRIVE guidelines (ARRIVE guidelines 2.0).

### 2.2. LCWE Preparation

LCWE was prepared as described previously [23,24]. Briefly, *Lactobacillus casei* (ATCC 11578; American Type Culture Collection, Manassas, VA, USA) was cultured in MRS broth (BD Difco, Franklin Lakes, NJ, USA) at 37 °C for 48 h. The bacterial cultures were subsequently washed several times with phosphate-buffered saline (PBS), after which the pellet was resuspended (5 g wet weight in 15 mL of PBS). The suspension was sonicated for 2 h in a cooling dry ice/ethanol bath using a Q500 sonicator with a 3/4-inch probe at an amplitude ranging from 70 to 80% (Q Sonica LLC, Newtown, CT, USA). The lysate was centrifuged at 20,000× *g* for 1 h at 4 °C, and the supernatant containing the cell wall extract was collected. The concentration of LCWE was determined on the basis of the rhamnose content using a phenol–sulfuric acid colorimetric assay and adjusted to 5 mg/mL with PBS. For disease induction, the mice received an intraperitoneal injection of 1000 μg of LCWE (0.2 mL).

### 2.3. Experimental Protocol

After acclimation, five-week-old male C57BL/6J mice were randomly assigned to experimental groups using a simple randomization method. A total of 12 mice received an intraperitoneal injection of LCWE (1000 μg), and 2 weeks later, they were allocated into two groups (*n* = 6 per group):

• LCWE group: received normal drinking water.
• LCWE+ARB group: received drinking water containing losartan (100 mg/L; Sigma–Aldrich Co. LLC, St. Louis, MO, USA) for 14 weeks.

The control group (*n* = 5) received PBS instead of LCWE and was provided with normal drinking water on the same schedule. After LCWE injection, approximately equal numbers of mice were allocated to the LCWE and LCWE + ARB groups (*n* = 6 per group). Formal randomization and allocation concealment procedures were not performed. The treatment allocation was concealed from the investigators responsible for histopathological assessment. No formal a priori sample size or power calculation was performed. Sample sizes were determined on the basis of our previous experience with the LCWE-induced vasculitis model while considering the principles of animal welfare and the 3Rs (replacement, reduction, and refinement). Accordingly, no single primary outcome was predefined for sample size determination. Histopathological evaluations were performed in a blinded manner by the investigators according to predefined scoring criteria and were not independently reviewed by a board-certified veterinary pathologist.

Losartan was dissolved in the drinking water at a concentration of 100 mg/L and administered ad libitum throughout the treatment period. On the basis of an average daily water intake of approximately 5 to 10 mL and a body weight of 25 g, this dosage ranged from approximately 20–40 mg/kg/day. To ensure the homogeneity of the solution and prevent drug precipitation, the drinking bottles were gently mixed once daily. Water consumption was monitored and did not differ from that of the control mice receiving regular drinking water. In addition, the drinking bottles were replaced with freshly prepared solutions once weekly. No animals were excluded from the analysis. At the end of the experimental period, the mice were euthanized under isoflurane anesthesia. Blood samples were collected by cardiac puncture under isoflurane anesthesia using a 1-mL syringe fitted with a 27-gauge needle. The needle was inserted into the beating heart through the thoracic wall, and blood was slowly aspirated after blood reflux was confirmed. Approximately 0.5–0.8 mL of blood was obtained from each mouse. Following blood collection, the hearts were immediately harvested for histological analysis. Humane endpoints, including significant weight loss (<20%), reduced mobility, or signs of distress, were monitored throughout the study; however, no animals reached these endpoints. The dose of losartan was selected on the basis of our previous study demonstrating its inhibitory effect on LCWE-induced coronary inflammation [15]. Young male C57BL/6J mice were used because KD shows a male predominance in humans [25] and because previous studies have demonstrated that compared with female mice, male mice develop more severe LCWE-induced vasculitis [26].

### 2.4. Histological Preparation

After euthanasia, the hearts were immediately excised and fixed in 20% neutral buffered formalin for 48 h with gentle agitation. Following fixation, the samples were briefly rinsed in water and transected horizontally into upper and lower halves. The upper half containing the aortic root was placed in a cassette for formalin-fixed paraffin-embedded (FFPE) processing. The tissues were dehydrated in 100% ethanol for 3–4 h, cleared in xylene, and embedded in paraffin using an automated tissue processor. The specimens were embedded with the cut surface facing downward to obtain transverse sections from the horizontal cut surface. Paraffin blocks were trimmed using a microtome until the aortic valve became visible. Serial sections were then cut at a thickness of 2.5 μm. Twelve consecutive sections were collected, after which approximately 60 μm of tissue was discarded before another set of 12 consecutive sections was collected. This serial sectioning procedure was repeated until the coronary arteries reached their origins from the aorta.

### 2.5. Histological Evaluations

As previously described, the CA inflammation score was graded using a four-point scoring system: 0, no inflammatory cell infiltration; 1, inflammatory cells localized to the adventitia; 2, inflammatory cells extending into the intima and adventitia; and 3, inflammation involving all layers of the arterial wall (panvasculitis) [23]. The total inflammation score was calculated from 10 sections per animal, including five sections from each CA. Aortitis incidence and aortic inflammation scores were also evaluated using the same scoring system as that used for coronary arteritis. The aortic inflammation score was calculated as the sum of inflammation scores from five aortic sections per animal.

CA stenosis was evaluated using three parameters: the incidence of neointima (%), intimal thickness (μm), and the percentage of luminal stenosis (%). The incidence of neointima was defined as the proportion of animals exhibiting intimal thickening of the CA within each group. Intimal thickness and luminal stenosis were quantified using the following formula:

$$CA\;stenosis\;(\%)=\frac{Area\;within\;internal\;elastic\;lamina\;(IEL)-Luminal\;area)}{Area\;within\;IEL}\times \;100\;$$

Elastin disruption was assessed using a semiquantitative scoring system: 0, no disruption of the elastic lamina; 1, fewer than 10 disruptions of the elastic lamina; 2, more than 10 disruptions of the elastic lamina; and 3, marked weakening or disappearance of the elastic lamina. Elastin disruption was defined as the interruption of the elastic lamina followed by the reappearance of the laminar structure. The number of interruptions was counted across five consecutive sections. Both the internal elastic lamina (IEL) and the external elastic lamina (EEL) of the bilateral CA were evaluated. Morphometric measurements were performed using NIS-Elements AR software (version 5.11.00; Nikon Instruments Inc., Tokyo, Japan).

### 2.6. Immunohistochemistry

Formalin-fixed, paraffin-embedded cardiac tissue sections (2.5 μm thick) obtained from experimental mice were immunostained using the following primary antibodies: anti-α-SMA (1:1000, clone 1A4, DAKO, Glostrup, Denmark), anti-PCNA (1:800, ab18197, Abcam, Cambridge, UK), anti-calponin (1:1000, ab46794, Abcam), and anti-MMP-9 (1:400, c-21733, Santa Cruz Biotechnology, Dallas, TX, USA). For each staining procedure, isotype control sections processed without the primary antibody were included and showed no specific staining. α-SMA expression was quantified by counting positively stained cells in both the intima and media. PCNA expression was quantified in the intima only, whereas calponin expression was assessed in the media only.

### 2.7. Measurement of Serum Cytokine Levels by ELISA

Serum samples were obtained from mice by direct cardiac puncture under isoflurane anesthesia at the time of euthanasia. The collected blood was centrifuged to obtain serum, and the samples were stored at −80 °C until analysis. Serum levels of matrix metalloproteinase-9 (MMP-9), tissue inhibitor of metalloproteinases-1 (TIMP-1), platelet-derived growth factor-AA (PDGF-AA), and transforming growth factor-β (TGF-β) were measured using multiplex immunoassays. MMP-9 and PDGF-AA levels were quantified using the Luminex Mouse Discovery Assay (Cat. #F-RD-LuminexMM-02). TGF-β was measured using the MILLIPLEX Multi-Species TGF-β1 Panel Single-Plex (Cat. #F-MIL-TGFBMAG-64K-01). TIMP-1 expression was measured using the Luminex Mouse Discovery Assay (1-Plex) (Cat. #F-RD-LuminexMM-01). All measurements were performed by an external laboratory (Filgen Inc., Aichi, Japan) in accordance with the manufacturer’s instructions. The concentrations of all the analytes are expressed in pg/mL.

### 2.8. Statistical Analysis

Continuous variables are expressed as medians and interquartile ranges (IQRs), and categorical variables are expressed as numbers and percentages (%). Normality was assessed using the Shapiro–Wilk test. Because several variables did not follow a normal distribution, comparisons among the three groups were performed using the Kruskal–Wallis test followed by Dunn’s multiple comparisons test. Categorical variables were compared using the chi-square test or Fisher’s exact test, as appropriate. All the statistical tests were two-tailed, and *p* < 0.05 was considered to indicate statistical significance. Statistical analyses were performed using SPSS version 24.0 (IBM Corp., Armonk, NY, USA) and GraphPad Prismversion 9.5.1 (GraphPad Software, San Diego, CA, USA).

## 3. Results

**Figure 1:**
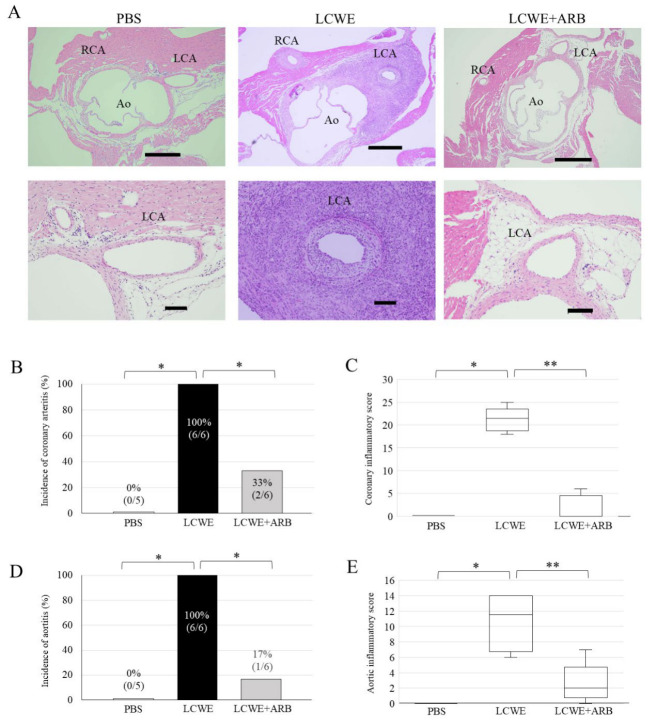
Anti-inflammatory effects of losartan on LCWE-induced coronary arteritis. (**A**) Representative hematoxylin and eosin (H&E)-stained cross sections of the aortic root, including bilateral coronary arteries from phosphate-buffered saline (PBS)-injected mice (**left**), *Lactobacillus casei* cell wall extract (LCWE)-injected mice (**middle**), and LCWE-injected mice treated with angiotensin receptor blocker (ARB) (**right**). Bar graphs show the incidence of coronary arteritis (**B**) and aortitis (**D**), as well as the inflammatory scores of the coronary arteries (**C**) and aortas (**E**), in PBS-, LCWE-, and LCWE+ARB-treated mice. Scale bars: 500 μm in the upper panels and 100 μm in the lower panels. Continuous variables are presented as medians and interquartile ranges (IQRs) in each group (PBS: *n* = 5; LCWE: *n* = 6; LCWE + ARB: *n* = 6). Comparisons among groups were performed using Fisher’s exact test for the incidence of coronary arteritis (**B**) and aortitis (**D**) and the Kruskal–Wallis test followed by Dunn’s multiple comparisons test for the coronary artery inflammatory score (**C**) and aortic inflammatory score (**E**). * *p* < 0.05 and ** *p* < 0.01. PBS, phosphate-buffered saline; LCWE, *Lactobacillus casei* cell wall extract; ARB, angiotensin receptor blocker; RCA, right coronary artery; LCA, left coronary artery; Ao, aorta.

The effects of losartan on LCWE-induced coronary arteritis are shown in Figure 1. Representative hematoxylin and eosin (H&E)-stained sections of the aortic root, including bilateral coronary arteries, revealed severe panvasculitis involving both the aortas and coronary arteries in LCWE-treated mice, whereas inflammatory cell infiltration was markedly attenuated in the LCWE+ARB group (Figure 1A). The incidence of coronary arteritis and aortitis was 100% in the LCWE group. In contrast, ARB treatment significantly reduced the incidence of coronary arteritis to 33% and that of aortitis to 17% (both *p* < 0.05; Figure 1B,D). Furthermore, the CA inflammatory score was significantly greater in LCWE-treated mice than in PBS-treated mice (median [IQR]: 0 [0–0] vs. 21.5 [15–24.3], *p* = 0.001), whereas compared with the LCWE treatment, losartan treatment significantly reduced the inflammatory score (median [IQR]: 0 [0–9.5], *p =* 0.003 vs. LCWE group) (Figure 1C). Similarly, the aortic inflammatory score was significantly lower in the LCWE+ARB group than in the LCWE group (Figure 1E).

**Figure 2:**
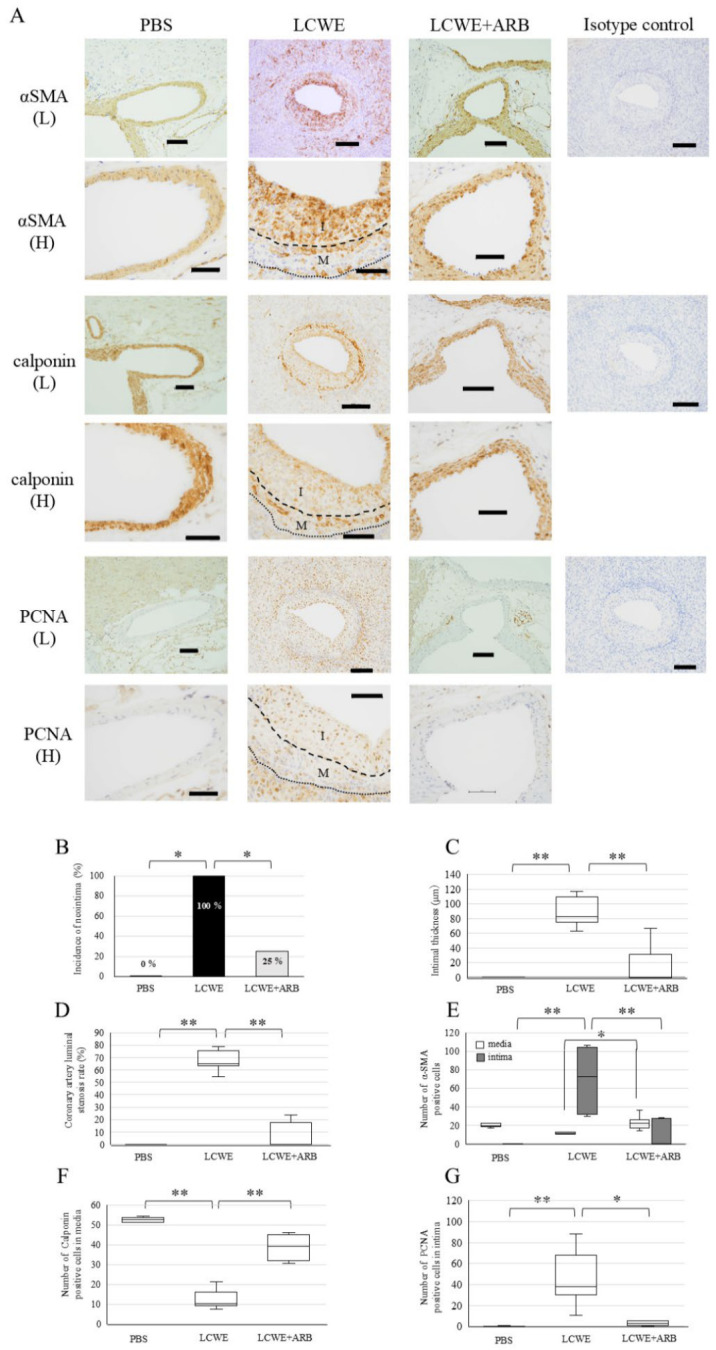
Immunohistochemistry of coronary artery stenosis. (**A**) Representative immunohistochemical staining for α-smooth muscle actin (α-SMA), calponin, and proliferating cell nuclear antigen (PCNA) in coronary artery cross-sections from phosphate-buffered saline (PBS)-injected mice (**left**), *Lactobacillus casei* cell wall extract (LCWE)-injected mice (**middle**), and LCWE-injected mice treated with angiotensin receptor blocker (ARB) (**right**). Corresponding isotype control sections are shown in the far-right panels. Scale bars: 100 μm in the low-magnification (L) panels and 50 μm in the high-magnification (H) panels. Thick dotted lines indicate the internal elastic lamina (IEL), whereas fine dotted lines indicate the external elastic lamina (EEL). Incidence of neointima (**B**), intimal thickness (**C**), coronary artery luminal stenosis rate (**D**), number of α-SMA-positive cells (**E**), number of calponin-positive cells in media (**F**), and number of PCNA-positive cells in intima (**G**) were compared among the three groups. Data are presented as the medians and interquartile ranges (IQRs) in each group (PBS: *n* = 5; LCWE: *n* = 6; LCWE+ARB: *n* = 6). Comparisons among groups were performed using Fisher’s exact test for the incidence of coronary stenosis (**B**) and the Kruskal–Wallis test followed by Dunn’s multiple comparisons test for intimal thickness (**C**), coronary artery luminal stenosis rate (**D**), α-SMA-positive cell counts in the intima and media (**E**), calponin-positive cell counts in the media (**F**), and PCNA-positive cell counts in the intima (**G**). * *p* < 0.05, ** *p* < 0.01. PBS, phosphate-buffered saline; LCWE, *Lactobacillus casei* cell wall extract; ARB, angiotensin receptor blocker; H&E, hematoxylin and eosin; α-SMA, α-smooth muscle actin; PCNA, proliferating cell nuclear antigen; I, intima; M, media; (L), low magnification; (H), high magnification.

Representative immunohistochemical staining demonstrated marked neointimal thickening with increased α-SMA-positive cells in the intima of LCWE-treated mice, whereas these changes were attenuated in the LCWE+ARB group (Figure 2A). In contrast, calponin-positive cells in the medial layer markedly decreased in the LCWE group but were relatively preserved following ARB treatment. PCNA-positive proliferating cells were predominantly observed in the neointima of LCWE-treated mice and were reduced in the LCWE+ARB group.

The incidence of coronary stenosis was 100% in the LCWE group and was significantly reduced to 25% in the LCWE+ARB group (*p* < 0.05; Figure 2B). Quantitative analysis revealed significant reductions in intimal thickness and the luminal stenosis rate in the LCWE+ARB group compared with those in the LCWE group (Figure 2C,D). In addition, the number of α-SMA-positive cells in the intima was significantly decreased by ARB treatment, whereas the number of medial calponin-positive cells was significantly preserved (Figure 2E,F). The number of PCNA-positive proliferating cells in the intima was also significantly lower in the LCWE+ARB group than in the LCWE group (Figure 2G).

**Figure 3:**
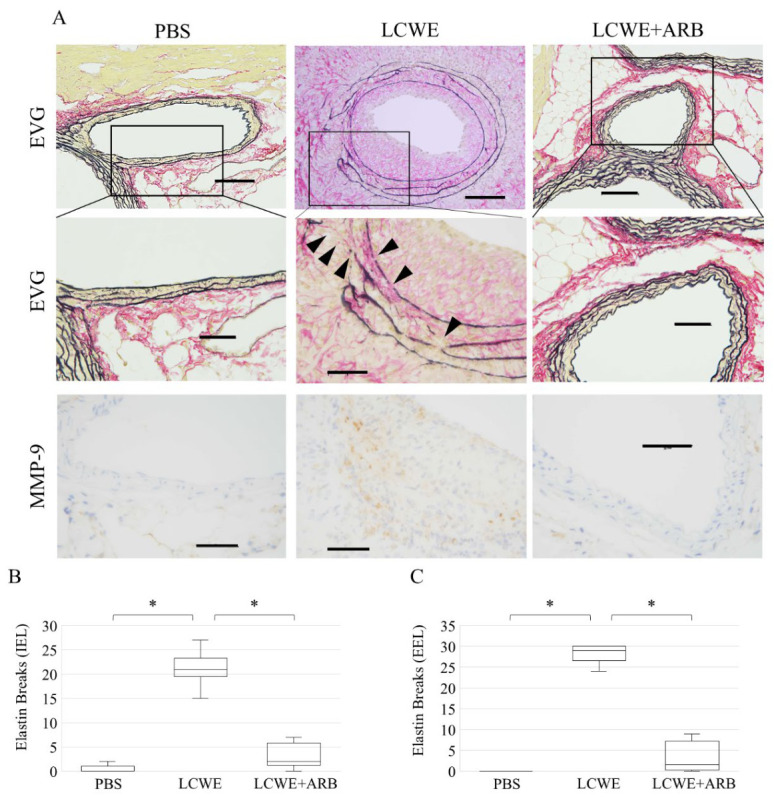
Effect of losartan on LCWE-induced elastin degradation. (**A**) Representative Elastica van Gieson (EVG)-stained cardiac tissue sections from mice treated with phosphate-buffered saline (PBS), *Lactobacillus casei* cell wall extract (LCWE), or LCWE-treated mice receiving an angiotensin receptor blocker (ARB). Enlarged views of the boxed regions are shown in the middle panels. Arrowheads indicate sites of elastic fiber fragmentation in LCWE-injected mice. The lower panels show high-magnification images of matrix metalloproteinase-9 (MMP-9) staining. In LCWE-treated mice, increased MMP-9 expression (brown staining) was observed in regions corresponding to sites of elastic fiber fragmentation. Quantification of elastin breaks in the internal elastic lamina (IEL) (**B**) and external elastic lamina (EEL) (**C**) in each group (PBS: *n* = 5; LCWE: *n* = 6; LCWE+ARB: *n* = 6). Scale bars: 100 μm (upper panels) and 20 μm (middle and lower panels). Data are presented as medians and interquartile ranges (IQRs). Comparisons among groups were performed using the Kruskal–Wallis test followed by Dunn’s multiple comparisons test for the IEL elastin break score (**B**) and EEL elastin break score (**C**). * *p* < 0.001. PBS, phosphate-buffered saline; LCWE, *Lactobacillus casei* cell wall extract; ARB, angiotensin receptor blocker; EVG, Elastica van Gieson; MMP-9, matrix metalloproteinase-9; IEL, internal elastic lamina; EEL, external elastic lamina.

Representative Elastica van Gieson (EVG) staining demonstrated marked disruption of vascular wall architecture in LCWE-treated mice, characterized by fragmentation of both the internal elastic lamina (IEL) and the external elastic lamina (EEL) (Figure 3A). In contrast, elastic fiber disruption was markedly attenuated in the LCWE+ARB group. Quantitative analysis revealed that the elastin break scores of both the IEL and EEL were significantly greater in LCWE-treated mice than in PBS-treated mice (median [IQR]: IEL, 1 [0–1.5] vs. 21 [18–24.8], *p* < 0.001; EEL, 0 [0–0.5] vs. 29 [25.5–30], *p* < 0.001) (Figure 3B,C). In contrast, losartan treatment significantly reduced elastin fragmentation, with median IEL and EEL scores decreasing to 2 [0.8–9.8] and 1.5 [0–13.3], respectively (both *p* < 0.001 vs. the LCWE group).

**Figure 4:**
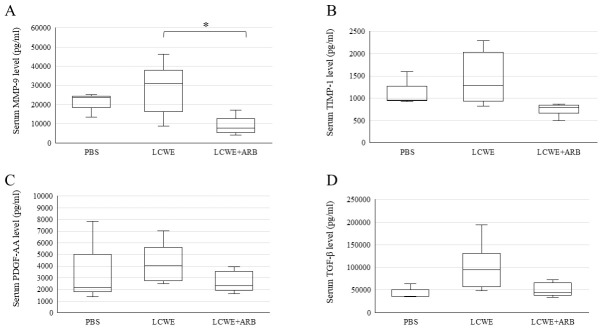
Serum concentrations of MMP-9 (**A**), TIMP-1 (**B**), PDGF-AA (**C**), and TGF-β1 (**D**). Data are presented as medians and interquartile ranges (IQRs) (PBS: *n* = 5; LCWE: *n* = 6; LCWE+ARB: *n* = 6). Comparisons among groups were performed using the Kruskal–Wallis test followed by Dunn’s multiple comparisons test. * *p* = 0.029. MMP-9, matrix metalloproteinase-9; TIMP-1, tissue inhibitor of metalloproteinase-1; PDGF-AA, platelet-derived growth factor-AA; TGF-β1, transforming growth factor-β1.

Quantitative analysis demonstrated that serum MMP-9 levels were significantly lower in the losartan-treated group than in the LCWE group (*p* = 0.029; Figure 4A). In contrast, serum TIMP-1 levels tended to decrease in the LCWE+ARB group, although the difference was not statistically significant (Figure 4B). Notably, the MMP-9/TIMP-1 ratio did not significantly differ among the groups. No significant differences in serum PDGF-AA or TGF-β levels were detected among the three groups (Figure 4C,D).

## 4. Discussion

First, losartan significantly suppressed coronary arteritis in the LCWE-induced murine model, supporting its anti-inflammatory effects in coronary vasculitis. Consistent with our previous study showing the attenuation of acute coronary perivasculitis and myocarditis and systemic inflammation in the same model [15], the present findings agree with experimental evidence that the inhibition of key inflammatory pathways, including the TNF-α and IL-1β signaling pathways, attenuated coronary arteritis in KD mouse models [27,28,29]. Because TNF-α contributes primarily to acute cardiac inflammation, whereas IL-1β is involved in subsequent coronary vasculitis, our findings suggest that suppressing persistent vascular inflammation may be beneficial even after disease onset.

Losartan markedly attenuated LCWE-induced CA stenosis. These findings are consistent with those of previous experimental studies showing that angiotensin II type 1 receptor blockade suppresses vascular restenosis and neointimal hyperplasia after balloon injury. Kauffman et al. demonstrated that losartan dose-dependently reduced neointimal thickening in rat carotid arteries following balloon injury [30], whereas Moon et al. reported that local delivery of losartan prevented recurrent stenosis, partly by inhibiting VSMC proliferation and migration [31]. In the present study, most neointimal cells were α-SMA positive, suggesting that VSMCs are the predominant cellular component of the neointima. We therefore evaluated histopathological changes associated with VSMCs during neointimal formation.

Losartan markedly reduced the number of intimal PCNA-positive cells and was associated with the preservation of calponin-positive medial VSMCs, suggesting reduced proliferative activity and maintenance of a contractile phenotype. Although direct evidence that losartan regulates VSMC phenotypic switching is lacking, previous studies have shown that losartan inhibits angiotensin II-induced VSMC proliferation, migration, and inflammatory activation [32,33,34]. In addition, preservation of the contractile VSMC phenotype has been associated with the suppression of neointima formation [35,36]. Our findings therefore suggest that losartan may attenuate CA stenosis while preserving a contractile medial VSMC phenotype. However, because the present study was limited to histopathological analyses, further in vitro studies and analyses of VSMC-related gene and protein expression are needed to clarify the underlying mechanisms involved.

In addition to its effects on VSMCs, inhibition of extracellular matrix degradation may also contribute to the anti-stenotic effects of losartan. MMP-9 promotes elastic fiber degradation, VSMC migration, and neointimal expansion [37,38,39,40] and is highly expressed in the coronary lesions of patients with KD [41]. Therefore, the reduction in MMP-9 levels observed in the present study may contribute to the preservation of vascular wall integrity and the attenuation of pathological vascular remodeling.

The protective effects of losartan observed in the present study are likely multifactorial. In addition to its antihypertensive action, losartan has been reported to exert anti-inflammatory and anti-remodeling effects by suppressing angiotensin II-mediated signaling, reducing inflammatory cell recruitment, attenuating extracellular matrix degradation, and preserving vascular integrity. In the present study, reduced coronary arteritis, decreased elastic lamina disruption, and lower serum MMP-9 levels following losartan treatment are consistent with these pleiotropic effects. However, the relative contributions of its anti-inflammatory, antihypertensive, and direct antiremodeling effects could not be distinguished in the current experimental design and warrant further investigation.

In the present study, losartan was administered at 100 mg/L in drinking water, corresponding to approximately 20–40 mg/kg/day in mice. Although this dose appears to be higher than the typical clinical pediatric dose on a mg/kg basis, direct comparison between mice and humans is difficult because of interspecies differences in drug metabolism, pharmacokinetics, and body surface area. Using body surface area–based conversion [42], the estimated human equivalent dose is approximately 1.6–3.2 mg/kg/day. Therefore, the present dosage should be interpreted as a pharmacological dose for preclinical proof-of-concept evaluation rather than a directly translatable clinical dose.

Losartan was selected for this study because we previously demonstrated its anti-inflammatory effects in a LCWE-induced vasculitis model [15] and because it has been reported to attenuate vascular remodeling and TGF-β signaling in several cardiovascular disease models, including atherosclerosis [43] and Marfan syndrome [44], suggesting potential translational relevance to KD-associated CA lesions. Future studies comparing different ARBs will be necessary to determine whether these beneficial effects are unique to losartan.

This study has several limitations. First, the relative contributions of the anti-inflammatory, antihypertensive, and direct antiremodeling effects of losartan to the inhibition of CA stenosis could not be distinguished in the present experimental design. Future studies using a delayed treatment protocol, initiated after the peak of coronary arteritis, may better clarify the direct antiremodeling effects of losartan. In particular, the direct effects of losartan on VSMC remodeling were not investigated and require further mechanistic studies. Second, blood pressure, cardiac function, and long-term cardiovascular outcomes were not evaluated, limiting our ability to determine the contribution of blood pressure reduction to the observed vascular protection. Third, the sample size was relatively small, although the number of animals was minimized in accordance with the 3R principles and ARRIVE guidelines. No formal prior power calculations were performed, and the relatively small sample size may limit the statistical power and generalizability of our findings. Fourth, although the LCWE-induced murine model reproduces several key pathological features of KD, it may not fully recapitulate the complex vascular pathology observed in human KD. Therefore, caution is warranted when these findings are extrapolated to clinical practice. Fifth, the histopathological evaluation was not independently reviewed by a qualified veterinary pathologist. This may have affected the robustness and interpretation of the histological findings and should be considered when interpreting the results. Finally, only male mice were included; therefore, potential sex-specific differences in the effects of losartan could not be assessed.

## 5. Conclusions

In this pilot study, losartan attenuated coronary artery stenosis in an LCWE-induced murine model of KD. These histopathological findings were associated with reduced neointimal formation, the preservation of medial calponin-positive VSMCs, a reduced number of PCNA-positive cells in the neointima, and lower serum MMP-9 levels. Although the underlying molecular mechanisms remain to be elucidated, these findings provide preliminary evidence supporting the potential role of losartan in attenuating vasculitis-associated coronary vascular remodeling. Further mechanistic studies are warranted to determine the potential translational relevance of these findings.

## Data Availability

Data are contained within the article.
